# Targeting Mutant p53 for Cancer Treatment: Moving Closer to Clinical Use?

**DOI:** 10.3390/cancers14184499

**Published:** 2022-09-16

**Authors:** Michael J. Duffy, Minhong Tang, Subhasree Rajaram, Shane O’Grady, John Crown

**Affiliations:** 1 UCD School of Medicine, Conway Institute of Biomolecular and Biomedical Research, University College Dublin, D04 V1W8 Dublin, Ireland; 2 UCD Clinical Research Centre, St. Vincent’s University Hospital, D04 T6F4 Dublin, Ireland; 3 Department of Medical Oncology, St Vincent’s University Hospital, D04 T6F4 Dublin, Ireland

**Keywords:** mutant p53, *TP53*, treatment, targeting, cancer, APR-246, statins, PC14586

## Abstract

**Simple Summary:**

Cancer is largely caused by genetic alterations such as mutations in a group of genes known as cancer driver genes. Many of the key advances in cancer treatment in recent years have involved blocking these driver genes using a new generation of anti-cancer drugs. Although p53 is the most frequently mutated gene in human cancers, historically, it has proved difficult to develop drugs against it. However, recently, several new drugs have become available for neutralizing the cancer-promoting effects of mutant p53. The aim of this article is to discuss the most promising of these drugs, especially those that are being investigated in clinical trials.

**Abstract:**

Mutant p53 is one of the most attractive targets for new anti-cancer drugs. Although traditionally regarded as difficult to drug, several new strategies have recently become available for targeting the mutant protein. One of the most promising of these involves the use of low molecular weight compounds that promote refolding and reactivation of mutant p53 to its wild-type form. Several such reactivating drugs are currently undergoing evaluation in clinical trials, including eprenetapopt (APR-246), COTI-2, arsenic trioxide and PC14586. Of these, the most clinically advanced for targeting mutant p53 is eprenetapopt which has completed phase I, II and III clinical trials, the latter in patients with mutant *TP53* myelodysplastic syndrome. Although no data on clinical efficacy are currently available for eprenetapopt, preliminary results suggest that the drug is relatively well tolerated. Other strategies for targeting mutant p53 that have progressed to clinical trials involve the use of drugs promoting degradation of the mutant protein and exploiting the mutant protein for the development of anti-cancer vaccines. With all of these ongoing trials, we should soon know if targeting mutant p53 can be used for cancer treatment. If any of these trials show clinical efficacy, it may be a transformative development for the treatment of patients with cancer since mutant p53 is so prevalent in this disease.

## 1. Introduction

Molecularly targeted therapy has had a major beneficial impact on the outcome of patients with several different cancer types in recent years. Thus, the use of drugs targeting HER2 amplification/overexpression in breast cancer [1], mutant EGFR in non-small cell lung cancer [2] and mutant BRAF in melanoma [3] has significantly improved the prognosis of patients with these respective cancer types. One highly prevalent cancer driver gene that remains to be successfully targeted for clinical utility is mutant *TP53* which encodes the p53 tumor suppressor protein.

*TP53* (p53) is the most widely studied gene in the human genome [4], with over 100,000 hits listed in PubMed. This intense investigation relates to the high prevalence of *TP53* mutations in most types of cancer. Indeed, across all human cancers, *TP53* is the most frequently altered gene with mutations occurring in approximately 50% of cases [5,6,7]. Furthermore, with a small number of exceptions (e.g., cutaneous melanoma), *TP53* is the most frequently mutated gene in most types of solid tumors. Most of these mutations belong to the missense type (70–80%) and are located in the DNA-binding region. Functionally, these mutations may result in loss of function, exertion of a dominant-negative effect or acquisition of a new ability [5,6,7]. Despite this high frequency, the presence of mutant p53 in tumors remains to be exploited and validated for clinical utility. This is especially true in the context of targeting mutant p53 for cancer treatment.

**Figure 1:**
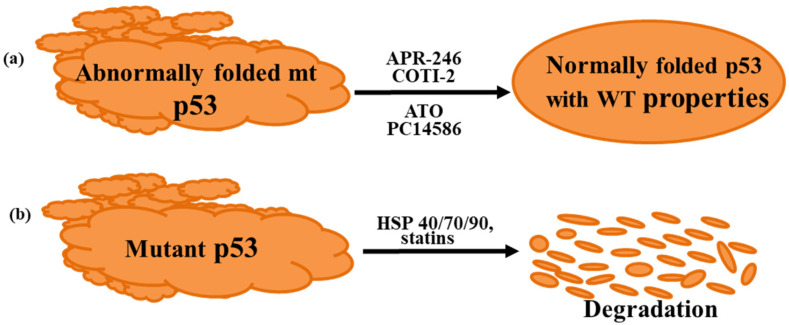
Strategies used to target mutant p53. (**a**) Reactivation of mutant p53 to a form with wild-type properties, (**b**) degradation and elimination of mutant p53. ATO, arsenic trioxide; HSP, heat shock protein.

Although there are currently no approved drugs for targeting mutant p53, several are currently undergoing clinical trials. Most of these investigational drugs mediate their action by either reactivating mutant p53 back to a form with at least some wild-type properties or by promoting degradation of the mutant protein [8,9] (Figure 1). The aim of this article is to review the emerging findings, focusing on the anti-p53 drugs currently in clinical trials. Firstly, however, we briefly discuss the multiple reasons that make mutant p53 a highly attractive target for drugs to treat cancer.

## 2. Mutant p53: A Highly Attractive Target for Cancer Treatment

### 2.1. High Prevalence in Cancer

As mentioned above, *TP53* mutations are present in approximately 50% of all cancers. Importantly, however, mutations occur at a particularly high prevalence (>80%) in some of the currently most aggressive tumors such as high grade serous ovarian cancer [10], triple negative breast cancer [11], esophageal cancer [12] and small cell lung cancer [13]. Thus, if an effective anti-mutant p53 agent was identified, it could have a major beneficial effect on the outcome of some of the cancer types that are currently difficult to treat.

### 2.2. Reactivation of Mutant p53 Can Potentially Induce Both Cell Intrinsic and Extrinsic Anti-Tumor Effects

Mutant p53 is believed to promote cancer development via two main mechanisms, i.e., cell intrinsic (direct effect on tumor cells) and cell extrinsic (direct effect on tumor microenvironment) [14,15,16]. Cell intrinsic effects include prevention of cell cycle arrest, enhancing proliferation, inhibition of apoptosis and blockage of DNA repair, while the main cell extrinsic effect is the promotion of immune evasion [14,15,16]. Thus, the reactivation of mutant p53 can potentially neutralize several different pro-cancer driving mechanisms including reactivation of host immunity against the cancer undergoing treatment.

### 2.3. Inhibition of Mutant p53 Function May Enhance Response to Standard Therapies

Available data suggest that several anti-cancer drugs act at least in part by promoting apoptosis mediated via wild-type p53 [17]. Consistent with this notion, findings from cell line and animal model studies show that the presence of mutant p53 confers resistance to a range of different therapies, including cytotoxic agents, targeted therapies and radiotherapy (for review, see refs. [17,18]). Reactivation of mutant protein to its wild-type configuration might therefore be expected to also confer sensitivity to these treatments. Supporting evidence for this possibility are the multiple reports showing that compounds that reactivate mutant p53 enhance response to several different clinically used therapeutics [18,19,20,21,22]. Another mechanism by which mutant p53 may confer drug resistance is by inducing expression of the drug efflux pump MDR1 (ABCB1), which promotes efflux of drugs out of tumor cells [23]. Again, reactivating mutant p53 back to its wild-type form should be expected to reverse this process, thus maintaining drug levels in the tumor cells.

### 2.4. Mutations in p53 Tend to Be Clonal

Clonal mutations, i.e., mutations that are present in all or most of the malignant cells within a tumor are believed to be superior targets for drug treatment than non-clonal mutations (mutations present in a subset of tumor cells). Based on available evidence, mutations in p53 appear to occur early and are clonal in many different cancer types [24].

### 2.5. Mutant p53 Proteins Accumulates in Malignant Cells

Mutant forms of p53 possessing missense mutations tend to accumulate in malignant cells. The accumulation may partly relate to the inability of the mutant protein to induce expression of the E3 ubiquitin ligase MDM2, which targets p53 for proteasomal-mediated catalyzed degradation [25,26]. In contrast, wild-type p53 promotes the expression of MDM2 in normal cells, thereby enhancing its degradation. Furthermore, mutant p53 is stabilized by interacting with different heat-shock proteins (HSP) which prevents its degradation by MDM2 and other E3 ubiquitin ligases [9]. Thus, levels of mutant p53 tend to be high in tumor cells but low in unstressed normal cells. This differential in levels of the p53 protein can result in a large therapeutic window, minimizing the possibility of therapy-related toxicity from mutant p53 targeting drugs.

## 3. Difficulties in Targeting Mutant p53

While theoretically, mutant p53 is a highly attractive target for cancer therapy, there are several difficulties in designing drugs to block its functioning. Indeed, it is considerably more challenging to restore normal activity to a defective tumor suppressor protein such as mutant p53 than to block the actions of a driver oncoprotein [27]. The difficulties in targeting mutant p53 are summarized in Table 1 and discussed in more detail below.

### 3.1. Multiplicity of Mutations with Different Structures and Functions

Unlike several oncogenes which are usually mutated at a small number of hot spot areas, p53 has been shown to undergo at least 2000 different mutations (giving rise to >2000 mutant/variant proteins) [28]. Indeed, over 85% of the amino acids in p53 have been found to be mutated, but no single mutation is found at a frequency of >6% [29,30]. Although approximately 90% of mutations in p53 are of the missense type, these are broadly divided into 2 main types, contact and structural [31]. Contact mutations allow the protein to largely maintain its wild-type conformation. On the other hand, conformation (also known as structural) mutations cause protein destabilization and unfolding at physiological temperatures. Both these types of mutation result in defective p53 binding to DNA which leads to defective transcription. Depending on the context of the malignant cell, this defective transcription can result in loss of wild-type function, exertion of a dominant-negative effect on the remaining wild-type allele or gain of an oncogenic property such as promotion of metastasis or resistance to specific drugs [16,32].

Further complicating the impact of the different mutations is that the same mutation might have different effects in different cell types, i.e., it is cell-context dependent. For example, p53 has been reported to induce the transcription of different sets of genes in different tissue types [33,34]. Indeed, in some situations, mutant p53 protein may not function as an oncogene but behave as a tumor suppressor gene [35]. For example, in a mouse model of intestinal cancer, Kadosh et al. [35] recently reported that a mutant form of p53 had different consequences in different parts of the gut. Thus, in the distal part, mutant p53 behaved as might be expected from a gene with oncogenic properties. In contrast, it acted as a tumor suppressor in the proximal gut. Interestingly, the tumor-suppressive effect of p53 was found to be eliminated by gallic acid produced by the gut microbiome.

Adding yet further diversity is that the mutant p53 protein can be post-translationally modified at multiple amino acid residue sites. These alterations, which include phosphorylation, acetylation, methylation, ubiquitylation and glycosylation [36], may also impact mutant p53 in several different ways such as altering stability and ability to interact with DNA or specific transcriptional factors [36].

Developing a specific inhibitor against all of the different forms of mutant p53 might be expected to be highly difficult, if not impossible.

### 3.2. Most Mutant Forms of p53 Lack a Suitable Pocket for High-Affinity Binding of Low Molecular Weight Compounds

With the exceptions of Y220C, Y220S and Y220N, most mutant forms of p53 lack an obvious deep pocket into which low molecular compounds can bind with high affinity and specificity [37]. Despite this, several low molecular weight compounds (e.g., specific mild alkylating chemicals) have been found to covalently bind to thiol groups in the core DNA binding domain of p53 [38,39,40,41] and result in reactivation of the mutant protein. Indeed, some of these compounds, such as eprenetapopt/methylene quinuclidinone (MQ) and arsenic trioxide (ATO) are currently undergoing clinical trials in patients with different types of cancer, see below.

### 3.3. Location of Mutant p53 in Tumor Cells Renders It Largely Inaccessible for Certain Types of Drugs

Monoclonal antibodies are amongst the most specific drugs used to treat cancer, but their current use is largely confined to targeting transmembrane proteins. However, as mutant p53 is primarily located in the cell nucleus, it cannot be readily reached with the current generation of these antibodies. A possible strategy for overcoming this problem, however, is to target processed mutant p53 bound to MHC proteins on the cancer cell membranes with so-called bispecific T-cell receptor mimic antibodies [42,43].

## 4. Mutant p53 Reactivating Drugs Undergoing Clinical Trials

### 4.1. Eprenetapopt/APR-246

By far, the most widely investigated mutant p53 reactivating drug is eprenetapopt (previously known as APR-246). As the mode of action of this compound has been previously discussed in detail [44,45,46], it will be only briefly reviewed here. To mediate its anti-cancer activity, eprenetapopt is first converted to MQ, which in turn binds to specific thiol groups in the p53 DNA-binding domain [38,39]. The specific thiol residues modified by MQ depends on whether p53 is present in a free state or attached to DNA [41]. Following MQ binding, mutant p53 undergoes refolding back to its wild-type configuration. In doing so, it regains its wild-type properties, such as the ability to induce apoptosis and inhibit cancer cell proliferation [44,45,46].

As might be expected from a small molecule that binds to thiol groups, eprenetapopt/MQ can also attach to other intracellular molecules containing these structures. In particular, it can also attach to the tripeptide, glutathione (GSH) and the redox modulating enzymes, thioredoxin reductase thioredoxin and glutaredoxin [47,48,49,50]. Binding to these reducing molecules can lead to increased levels of reactive oxygen species (ROS). Since cancer cells tend to have increased levels of ROS, they may be more likely than normal cells to undergo cell death in response to the enhanced increase mediated by eprenetapopt/MQ [51]. The increased production of ROS, can in turn, potentially enhance the anti-cancer activity of eprenetapopt. In addition, the high concentration of ROS may lead to the degradation of mutant p53, potentially further enhancing the anti-cancer effects of eprenetapopt [52].

Based on several preclinical studies showing anti-cancer activity [8,44], eprenetapopt was investigated for potential toxicity in a phase I clinical trial [53]. This small trial involved patients with acute myeloid leukemia (AML) (*n* = 7) or prostate cancer (*n* = 7). Overall, eprenetapopt was found to be well tolerated, the most frequent adverse effects were fatigue, dizziness, headache and confusion, all of which were reported to be reversible. Evidence for reactivation of mutant p53 was the finding of cell cycle arrest, induction of apoptosis and upregulation of p53 target genes in tumor cells from several treated patients.

Subsequently, eprenetapopt in combination with azacytidine was investigated in a phase Ib/II trial in patients with *TP53* mutant myelodysplastic syndromes (MDS) (*n* = 40) or AML (*n* = 11) [54]. The overall response rate was 71% with 44% of the treated patients achieving a complete response. For patients with MDS, 29 (73%) responded with 20 (50%) having a complete response and 23 (58%) a cytogenetic response. In the patients with AML, 7 (64%) exhibited an overall response while 4 (36%) had a complete response. Interestingly, patients with *TP53* mutations exhibited a significantly higher complete response rate than those lacking mutations (69% vs 25%; *p* = 0.006). Furthermore, patients achieving a response had a reduction in both *TP53* mutation levels (i.e., variant allele frequency) and p53 protein levels. The most frequent adverse events (grade ≥ 3) in this trial were febrile neutropenia (33%), leukopenia (29%) and neutropenia (29%). Essentially similar findings were reported with eprenetapopt plus azacytidine in a second phase II trial involving patients with MDS or AML [55].

The above findings led to a phase III trial of eprenetapopt plus azacitidine versus azacitidine for the frontline treatment of patients with *TP53* mutant MDS. Although results from this trial do not appear to have been published to date in the peer-reviewed literature, according to the Aprea website (https://ir.aprea.com/node/7691/pdf, accessed on 1 June 2022), it failed to meet the primary statistical endpoint of complete remission. Complete response rates however, tended to be higher in the combination arm, i.e., 33.3% of patients receiving eprenetapopt with azacytidine achieved a CR versus 22.4% in the azacitidine alone arm. However, the press release stated that with further follow-up, secondary endpoints such as overall response rate and duration of response might favor the combination arm.

Despite this negative finding, eprenetapopt has received Breakthrough Therapy, Orphan Drug and Fast Track designations from the US FDA for MDS, and Orphan Drug designation from the European Commission for patients with MDS, AML and ovarian cancer.

### 4.2. COTI-2

COTI-2 is described as a third generation thiosemicarbazone drug with broad anti-cancer activity [56,57,58]. Compared to eprenetapopt, it has been less investigated both experimentally and clinically. However, similar to eprenetapopt, COTI-2 was found to change the mutant conformation of p53 and return it to a form with specific wild-type abilities [57,58]. Using the technique of surface plasmon resonance, COTI-2 was shown to bind to mutant p53 with high affinity, although the specific binding amino acid residues were not identified [57]. Furthermore, COTI-2 was reported to normalize wild-type p53 target gene expression and restore wild-type p53 binding to DNA [58].

Other actions of COTI-2 include activation of AMP-activated protein kinase (AMPK) and inhibition of mTOR signaling [58]. These effects may be secondary to reactivation of mutant p53 to its wild-type counterpart, as wild-type p53 has been shown to both inhibit mTOR and activate AMPK [59,60]. These effects of COTI-2 may further enhance the anti-cancer potential of the drug. Indeed, inhibition of mTOR with drugs such as everolimus is a well-established strategy for treating certain cancers such as estrogen-receptor-positive metastatic breast cancer [61].

COTI-2 as a monotherapy or in combination with standard therapies has undergone evaluation for the treatment of several different types of recurrent cancers in a phase I clinical trial (NCT02433626). According to the US National Library of Medicine, ClinicalTrial.gov website (https://clinicaltrials.gov/ct2/show/results/NCT02433626, accessed on 1 June 2022), this trial “is designed primarily to assess the safety and tolerability of COTI-2 monotherapy or combination therapy in patients with advanced and recurrent malignancies to establish a recommended phase II dose for future studies”. Preliminary data suggest that COTI-2 is well tolerated, the most frequent adverse effects being nausea, vomiting, fatigue and abdominal pain. Of the 24 patients treated, only 2 (8%) were reported to have to undergo a decrease in the dose of COTI2 administered [62]. So far, there appear to be no published data with respect to tumor regression in patients treated with COTI-2.

### 4.3. Arsenic Trioxide

Arsenic trioxide (ATO), a drug which has been used for several years to treat acute promyelocytic leukemia, was recently shown to reactivate mutant forms of p53 possessing structural mutations [63]. This activation resulted in the restoration of biological function and inhibition of tumor cell growth, both in vitro and in vivo [63]. In contrast to eprenetapopt, ATO did not appear to activate mutant p53 possessing contact mutations or induce the transcription of p53 target genes such as PUMA or CDKN1A in tumor cells with such mutations. Similar to eprenetapopt, however, ATO was found to bind to thiol residues in mutant p53 (C124, C135 and C141) [63]. Interestingly, the eprenetapopt metabolite, MQ was also found to bind to C124 when mutant p53 was attached to DNA [41].

In addition, a reactivating p53, ATO, has also been reported to degrade the mutant protein [64]. Thus, ATO appears to be able to neutralize the cancer promoting effects using two different mechanisms.

A potential advantage of ATO over other mutant p53-reactivating drugs such as eprenetapopt and COTI-2 is that its pharmaceutical and toxicological properties are well established [65]. It should therefore be highly suitable for repurposing for the treatment of other tumor types, especially those possessing structural p53 mutations. Indeed, recently, ATO began to be evaluated for efficacy, safety and tolerability in a phase I trial (PANDAtrial) involving patients with refractory ovarian and endometrial cancers possessing structural p53 mutations (ClinicalTrials.gov Identifier: NCT04695223).

### 4.4. PC14586

In contrast to the mutant p53 reactivating drugs discussed above, PC14586 specifically reactivates mutant p53 proteins containing the Y220C mutation [66]. As mentioned earlier in this article, this mutation creates a small pocket in the p53 protein, rendering it thermally unstable and unable to bind to DNA. This mutation, however, is relatively rare in human cancers, being present in only approximately 2% of all tumors [37]. Nevertheless, according to Fersht and colleagues [67], targeting the Y220C mutation is an ideal test case for the binding of potential low molecular weight inhibitors to mutant p53 as the tyrosine-cysteine mutation creates a specific surface pocket that is druggable with small molecules.

In mutant cell lines, PC14586 was found to reactivate and stabilize the p53 Y220C mutant protein [66]. In turn, this resulted in transcription of the p53 wild-type target genes, *BAX*, *PUMA*, *MDM2* and *CDKN1A* (encodes *p21*) as well as induction of cell cycle arrest. Consistent with the ability of mutant p53 to induce immunosuppression (see above), administration of PC14586 was found to enhance immunity by increasing influx of immune cell such as CD4+ T cells, CD8+ T cells, T-regulatory cells and natural killer T cells into tumors [68]. In mice models of gastric cancer possessing the Y220C, administration of PC14586 led to dose responsive anti-tumor effects, while in a C57Bl/6J syngeneic xenograft model possessing a Y220C mutation, administration of PC14586 was found to result in complete tumor regression in 80% of the treated mice [66].

PC14586 monotherapy is currently being investigated in a phase I/II clinical trial in patients with advanced cancers harboring the p53 Y220C mutation (NCT study identifier NCT04585750). The aim of this multicenter dose escalation study is to evaluate PC14586 safety, pharmacokinetics, pharmacodynamics and possibly efficacy in patients with advanced solid tumors possessing a p53 Y220C mutation. Preliminary findings presented at the 2022 American Society of Clinical Oncology conference suggest that PC14586 is generally well-tolerated with only grade I/II adverse events which were found in 79% of the treated patients [69]. The most frequent adverse events were nausea (34%), vomiting (24%), fatigue (21%) and increased aspartate aminotransferase activity (17%). Using RECIST v1.1 criteria, overall responses were observed in 8/25 (32%) of the patients treated with higher doses of the drug (i.e., ranging from 1150 mg daily to 1500 mg twice daily).

Recently, the US FDA granted Fast Track designation to PC14586 for the treatment of cancer patients with locally advanced or metastatic solid tumors possessing a *TP53* Y220C mutation.

The advantage of mutant-specific drugs such as PC14586 over other mutant p53 reactivators such as eprenetapopt is that they are unlikely to bind to wild-type p53 or indeed to other intracellular proteins. The main disadvantage, however, is that the p53 Y220C mutation is relatively rare in cancer, being only the 9th most frequent mutation. Despite this low frequency, the Y220C is estimated to be responsible for in excess of 100,000 cases of cancer per year, worldwide [37].

## 5. Mutant p53 Degrading Drugs Undergoing Clinical Trials

Another strategy for targeting “difficult to drug” driver oncoproteins such as mutant p53 is to treat with compounds that promote their degradation and thus their elimination from tumor cells. Such drugs in everyday clinical use include fulvestrant which acts by degrading the estrogen receptor and is used for the treatment of patients with estrogen receptor-positive metastatic breast cancer [70] and lenalidomide which induces cereblon to degrade the transcription factors, IKZF1 and IKZF3 [71]. Lanalidomide is used in the treatment of multiple myeloma.

Proof of principle that the presence of mutant p53 was necessary for the maintenance of malignancy emerged from knockdown studies which showed that depletion of the mutant protein reduced oncogenicity [72]. Amongst the earliest pharmaceutical drugs shown to degrade mutant p53 protein were heat-shock protein (HSP) inhibitors [73]. To mediate oncogenic activity, mutant p53 must be stabilized and maintained in tumor cells [74]. As mentioned above, one of the main mechanisms of stabilization is achieved by binding with HSP such as HSP40, HSP70 and HSP90. HSPs appear to prevent mutant p53 degradation by blocking the binding of E3 ubiquitin ligases such as MDM2 or CHIP to the mutant protein [75,76]. Thus, inhibiting the interaction between a HSP and mutant p53 might be expected to lead to mutant p53 degradation and thus suppression of cancer growth.

These observations led to the testing of low molecular weight HSP inhibitors as potential drugs for cancer treatment. Early work using preclinical models confirmed that treatment with low molecular weight HSP90 inhibitors such as ganetespib, 17-allylamino-17-demethoxygeldanamycin or geldanamycin did indeed result in the degradation of specific forms of mutant p53 and inhibition of tumor cell growth [73]. In contrast to the findings in p53 mutant cells, the HSP inhibitors failed to block proliferation in p53-null cells [72]. However, in clinical trials, one of the most potent HSP90 inhibitors, i.e., ganestespib, was found to have unacceptable toxicity and to lack efficacy [77,78,79]. Thus, research into these compounds was largely abandoned.

More recently, treatment with a group of widely used drugs known as statins, was also shown to result in degradation of mutant p53 [80,81,82,83]. Statins are extensively used to treat high levels of cholesterol, especially the cholesterol bound to low density lipoprotein, thus helping to minimize the occurrence of heart disease and stroke. They decrease cholesterol levels by inhibition 3-hydroxy-3-methylglutaryl-CoA reductase (HMGCR), the rate-limiting enzyme in its formation. However, while lowering cholesterol levels, statins also decrease levels of multiple metabolites in the cholesterol biosynthetic pathway, including mevalonate phosphate (MVP) [84].

Several studies have recently shown that decreased production of MVP leads to the degradation of mutant p53 [80,81,82,83]. The decreased formation of MVP resulting from treatment with statins promotes the release of mutant p53 from HSPs which in turn leads to degradation of the mutant protein. In one of these studies, Parrales et al. [80] reported that treatment of malignant cells with the statin, lovastatin decreased the binding of p53 containing conformational mutations to a HSP40 isoform known as DNAJA1. This decreased binding to DNAJA1 led to the degradation of mutant p53 but not wild-type p53, mediated by the ubiquitin E3 ligase, CHIP. In a further report, treatment with the statin, cerivastatin was found to result in the dissociation of mutant p53 from HSP90 and degradation by MDM2 [81].

Consistent with their ability to degrade mutant p53, several different statins when used alone have been shown to inhibit the in vitro and in vivo growth of cancer cells in model systems [80,81,82,83,85]. Although lovostatin was reported to promote mutant p53 degradation only in tumor cells harboring *p53* with conformational mutations [80], other statins were shown to have inhibitory effects on cell lines independent of the type of p53 mutation [85].

Clinical evidence supporting an anti-cancer role for statins are multiple epidemiological studies showing that patients with a range of different cancer types undergoing treatment with statins have a superior outcome compared with those not receiving these drugs [84,86,87,88]. However, contradictory results have also been published [89]. Possible reasons for the conflicting results include the type of statin used (lipophilic statins being more readily taken up by cells than hydrophilic statins), dose administered, length of treatment, co-administered drug(s), whether the treatment was started before or after diagnosis and whether or not the tumor undergoing treatment contained p53 mutations (and possibly the type of mutation). It is important to state however, that there is currently a lack of data from randomized clinical trials demonstrating that patients with cancer receiving statins have an enhanced outcome compared to those not receiving the drug. However, at least three such clinical trials that include the prior measurement of the p53 mutational status/protein levels are currently ongoing.

One of these is a window-of-opportunity trial to determine if administration of atorvastatin decreases levels of p53 with conformational mutations in patients with different solid tumor or AML (NCT03560882). Another is a phase II trial investigating the effect of atorvastatin for treating patients with ulcerative colitis who have a dominant-negative missense *TP53* mutation and are at risk of developing large intestinal cancer (ClinicalTrials.gov Identifier: NCT04767984). A third trial (also a phase II trial) is investigation the benefit of neoadjuvant atorvastatin plus zoledronate in patients with triple negative breast cancer (YAPPETIZER; ClinicalTrials.gov Identifier: NCT03358017). In this study, response is being related to the level of cellular p53. In addition to these trials, several studies are investigating if statins have anti-cancer activity irrespective of p53 status (https://clinicaltrials.gov/ct2/results?cond=&term=statins&cntry=&state=&city=&dist=) (accessed on 1 June 2022).

Should any of these trials produce positive findings, statins might be expected to rapidly enter clinical use for the treatment of cancer. As with ATO (see above), statins have been in clinical use for several years. Thus, their pharmaceutical and toxicological properties are also well established. Since they are relatively safe, inexpensive and off-patent, they would be ideally suitable for repurposing for the treatment of cancer.

Before concluding this section, it is also important to add that in addition to degrading mutant p53 via downregulation of the mevalonate pathway, there are several other mechanisms by which statins could exert anti-cancer activity (for review, see ref. [90]). Indeed, the lowering of cholesterol levels per se by statins may have anti-cancer activity [91]. For example, in a recent report, Wang et al. [92] showed that use of a non-statin drug (ezetimibe) to reduce cholesterol levels resulted in enhanced anti-tumor immunity in several different mouse tumor models. It would be interesting to investigate if statins might also promote anti-tumor immunity.

## 6. Exploiting Mutant p53 for Vaccination

A further strategy for targeting mutant p53 that has progressed to clinical trial investigations is vaccination against the mutant protein. The aim of therapeutic cancer vaccination is to stimulate a patient’s adaptive immune system against tumor (neo)antigens and thus block tumor growth. As *TP53* is mutated in a large proportion of human cancers, it would be expected to act as a neoantigen and promote an immune response, thereby opening up the potential of exploiting it as an anti-cancer vaccine. Evidence from mice models and patients with cancer showed that administration of certain p53 neo-epitopes containing hot-spot mutations did indeed provoke the generation of specific T cell (CD8+ and CD4+ cells) responses against p53 peptides containing the hot-spot mutations [93,94]. Thus, cytotoxic T lymphocytes recognizing the p53_25–35_, p53_110–124_, p53_149–157_ and p53_264–272_ have been observed in experimental systems [93,94]. These findings led to the development of several different vaccines for enhancing T cell responses against tumors possessing mutant p53 [93,94]. Since these vaccines were generally well tolerated when administered to mice models, they were tested in multiple phase I/II clinical trials across different cancer types [93]. Although some of these vaccines promoted a p53-specific immune response, little clinical activity has been observed to-date [93].

Before concluding this section, it should be stated that these negative clinical findings are not unique to mutant p53 as a cancer vaccine [95]. Despite the enormous amount of research devoted to the development of anti-cancer vaccines, only two have so far been approved for clinical application, i.e., Sipuleucel T for advanced prostate cancer and talimogene laherparepvec (T-VEC) for the treatment of melanomas. This failure appears to be at least partly due to a lack of sufficient immunogenicity in the vaccines tested for inducing an appropriate clinical response for tumor elimination [95].

Future research on anti-cancer vaccines including p53 vaccines should therefore focus on optimizing delivery of the vaccine to the appropriate cellular location, selecting the optimum adjuvant to be co-administered with the vaccine, identifying the optimum dose and treating patients with early cancer (where immunosuppression would be expected to be limited) rather that patients with advanced cancer (where immunosuppression would be expected to be strong).

## 7. Other Strategies for Targeting Mutant p53

Several other strategies for targeting mutant p53 are currently undergoing investigations, most of which are still at a preclinical level (Table 2). Gene therapy with Gendicine (a recombinant human adenovirus containing wild-type p53) in combination with radiotherapy, however, has been approved for the treatment of head and neck squamous cancer in China but apparently not in any other country. This approval followed several clinical trials reporting that Genidicine was well tolerated and improved outcome in patients with advanced head and neck cancers (as well as in other cancer types [96]).

## 8. Targeting Mutant p53 Looking to the Future

Going forward, one of the most promising approaches for targeting mutant proteins such as mutant p53 is likely to be the use of bispecific T-cell receptor mimic antibodies, i.e., antibodies in which one end binds to a mutant epitope on a tumor cell and the other end to a T-cell. It was mentioned above that the present generation of monoclonal antibodies cannot readily access nuclear proteins such as mutant p53. However, peptides derived from such mutant proteins can be processed by the proteasome system and converted into small peptides. These peptides can attach to HLA proteins located on the cell membrane and hence be recognized by T-cell via their T-cell receptors.

To target such peptides, Hsiue et al. [42] developed an antibody specific for one of the most frequently occurring mutations in the *TP53* gene, i.e., the R175H mutation. The bispecific single chain antibody, named H2scD, bound to both the specific mutant p53 peptide attached to an HLA allele (A*02:01) and the T-cell receptor. By binding to both sites, the bispecific antibody was able to activate T cells and consequently eliminate cancer cells possessing the specific mutant peptide. Theoretically, this strategy could be used to target other mutant forms of p53 as well as other mutant proteins such as KRAS [42]. Although the use of single chain antibodies such as the type used in this report is promising for targeting mutant proteins, because of its relatively small size, it is likely to be rapidly cleared by the kidneys. Thus, methods to stabilize the antibody may be necessary.

Another potential strategy for targeting cancer-specific mutations is the use of CRISPR/Cas9 gene editing. This technology uses genetic engineering to alter the cells’ genome [102]. In one of the most successful attempts to date to target mutant p53 using CRISPR, Sayed, et al. [103] used the recently introduced adenine base system to target a pancreatic cancer cell line (PANC-1) expressing the *TP53* mutation, R273H. Evidence of gene correction was the induction of several canonical p53 target proteins such as p21, MDM4, PUMA and GADD45A. Further work by Sayed et al. [103] showed that this methodology also successfully corrected the *TP53* R175H mutation in a cell line derived from a colorectal cancer organoid. The challenge will be to translate this technology to patients with cancer.

Other potential strategies for targeting muting mutant p53 include the use of PROTACs/molecular glues which can lead to degradation of specific mutant proteins [101], induction of therapeutic hypothermia in tumors possessing temperature sensitive *TP53* mutations [100] and exploitation of synthetic lethality for cancer containing gain of function mutations [99]. Although all the above-mentioned strategies for targeting mutant p53 are promising, they still require optimization and further validation prior to any clinical trial

## 9. Conclusions

With the multiplicity of ongoing clinical trials, we should soon know whether targeting mutant p53 has clinical efficacy in cancer. The recent demonstration of response to PC14586 provides some optimism that mutant p53 may eventually be targeted for cancer treatment. However, irrespective of the outcome of the ongoing trials, mutant p53 remains a highly attractive target for cancer treatment (see above). We, therefore, encourage academic researchers and pharma companies to intensify their research into exploiting the most frequently mutated gene in cancer for therapeutic potential. The ultimate reward could be a drug with wide application across a broad range of cancer types.

## Figures and Tables

**Table 1:** Reasons why mutant p53 is difficult to target.

Multiplicity of mutations with different structures and functions
Absence of a readily identifiable pocket suitable for binding of drugs *
Absence of enzyme activity which might be blocked by catalytic inhibitors
Predominantly nuclear localization prevents access by standard antibodies

* the Y220C mutant form of p53 is an exception as it contains a binding pocket.

**Table 2:** Different strategies for targeting mutant p53 for cancer therapy.

Strategy	Example of Drug	Refs.
Reactivation to WT form	Eprenetapopt, COTI-2, arsenic trioxide, PC14586	[8,44,46,57,58,63,66]
Degradation of mt 53	Ganetespib, statins	[73,80,81,82,83]
Vaccines targeting mt p53	p53-SLP, p53MVA	[93,94]
Gene therapy	Gendicine *	[96]
P53 mt-specific antibodies	anti-R248Q antibody	[97]
P53 mt-specific siRNAs		[98]
T-cell receptor mimic antibodies	H2-scDb	[42]
Inhibiting genes exhibiting synthetic lethality with mt p53	*WEE1*, *ATR*, *CHK1*	[99]
Induction of therapeutic hypothermia via temperature sensitive mt forms of p53	CHA	[100]
Molecular glues/PROTACS	Manumycin polyketides	[101]
CRISPR/Cas9		[102,103]

* recombinant human p53 adenovirus; WT, wild-type; mt, mutant; p53-SLP, synthetic long; CHA, N6-cyclohexyladenoxine; PROTACS, proteolysis targeting chimeric.
